# Accuracy of prenatal and postnatal biomarkers for estimating gestational age: a systematic review and meta-analysis

**DOI:** 10.1016/j.eclinm.2024.102498

**Published:** 2024-03-08

**Authors:** Elizabeth Bradburn, Agustin Conde-Agudelo, Nia W. Roberts, Jose Villar, Aris T. Papageorghiou

**Affiliations:** a Nuffield Department of Women’s & Reproductive Health, University of Oxford, Oxford, UK; b Oxford Maternal & Perinatal Health Institute, Green Templeton College, University of Oxford, Oxford, UK; c Bodleian Health Care Libraries, University of Oxford, Oxford, UK

**Keywords:** Pregnancy, Gestational age, Screening, Growth, Preterm, Diagnostic accuracy, Metabolomics, Hormones

## Abstract

**Background:**

Knowledge of gestational age (GA) is key in clinical management of individual obstetric patients, and critical to be able to calculate rates of preterm birth and small for GA at a population level. Currently, the gold standard for pregnancy dating is measurement of the fetal crown rump length at 11–14 weeks of gestation. However, this is not possible for women first presenting in later pregnancy, or in settings where routine ultrasound is not available. A reliable, cheap and easy to measure GA-dependent biomarker would provide an important breakthrough in estimating the age of pregnancy. Therefore, the aim of this study was to determine the accuracy of prenatal and postnatal biomarkers for estimating gestational age (GA).

**Methods:**

Systematic review prospectively registered with PROSPERO (CRD42020167727) and reported in accordance with the PRISMA-DTA. Medline, Embase, CINAHL, LILACS, and other databases were searched from inception until September 2023 for cohort or cross-sectional studies that reported on the accuracy of prenatal and postnatal biomarkers for estimating GA. In addition, we searched Google Scholar and screened proceedings of relevant conferences and reference lists of identified studies and relevant reviews. There were no language or date restrictions. Pooled coefficients of correlation and root mean square error (RMSE, average deviation in weeks between the GA estimated by the biomarker and that estimated by the gold standard method) were calculated. The risk of bias in each included study was also assessed.

**Findings:**

Thirty-nine studies fulfilled the inclusion criteria: 20 studies (2,050 women) assessed prenatal biomarkers (placental hormones, metabolomic profiles, proteomics, cell-free RNA transcripts, and exon-level gene expression), and 19 (1,738,652 newborns) assessed postnatal biomarkers (metabolomic profiles, DNA methylation profiles, and fetal haematological components). Among the prenatal biomarkers assessed, human chorionic gonadotrophin measured in maternal serum between 4 and 9 weeks of gestation showed the highest correlation with the reference standard GA, with a pooled coefficient of correlation of 0.88. Among the postnatal biomarkers assessed, metabolomic profiling from newborn blood spots provided the most accurate estimate of GA, with a pooled RMSE of 1.03 weeks across all GAs. It performed best for term infants with a slightly reduced accuracy for preterm or small for GA infants. The pooled RMSEs for metabolomic profiling and DNA methylation profile from cord blood samples were 1.57 and 1.60 weeks, respectively.

**Interpretation:**

We identified no antenatal biomarkers that accurately predict GA over a wide window of pregnancy. Postnatally, metabolomic profiling from newborn blood spot provides an accurate estimate of GA, however, as this is known only after birth it is not useful to guide antenatal care. Further prenatal studies are needed to identify biomarkers that can be used in isolation, as part of a biomarker panel, or in combination with other clinical methods to narrow prediction intervals of GA estimation.

**Funding:**

The research was funded by the 10.13039/100000865Bill and Melinda Gates Foundation (INV-000368). ATP is supported by the Oxford Partnership Comprehensive Biomedical Research Centre with funding from the NIHR Biomedical Research Centre funding scheme. The views expressed are those of the authors and not necessarily those of the UK National Health Service, the NIHR, the Department of Health, or the Department of Biotechnology. The funders of this study had no role in study design, data collection, analysis or interpretation of the data, in writing the paper or the decision to submit for publication.

**Research in context**

**Evidence before this study**

Gestational age (GA) estimation is essential for optimal maternity care, but is often inaccurate, particularly in low- and middle-income countries (LMICs). Existing methods, such as last menstrual period (LMP), symphysis-fundal height (SFH), and late trimester ultrasound have limitations, including inaccuracy and the gold standard, first-trimester ultrasound, is often unavailable in LMICs. As a result, GA is unknown in a large proportion of women worldwide. This review explores potential biomarkers to enhance GA estimation.

A comprehensive search of databases, including MEDLINE, EMBASE, CINAHL, LILACS, medRxiv, Science Citation Index and other databases was conducted with no language restrictions from inception to September 15, 2023, employing terms related to GA, estimation, and biomarkers. The inclusion criteria encompassed studies reporting biomarker accuracy during the antenatal or immediate postnatal period, using ultrasound or LMP as comparators, and involving healthy mothers/newborns with relevant statistical assessments.

**Added value of this study**

This is the first systematic review of biomarkers for GA estimation, identifying promising candidates that could be useful in settings without early ultrasound access. Metabolomic profiling from heel-prick blood spot emerges as the most accurate, exhibiting a pooled RMSE of 1.03 weeks across all GAs, with optimal performance in term infants. Other biomarkers, such as cord blood metabolomic profiling and DNA methylation, exhibit lesser accuracy compared to newborn blood spot metabolomic profiling. During the prenatal period, hCG measured between 4 and 9 weeks had the highest correlation with the reference standard GA among the placental biomarkers assessed; however, this correlation was less accurate when assessed for a wider GA window. Two small studies suggested that maternal metabolomic profiling had low accuracy to estimate GA with a pooled RMSE of 2.90 weeks.

**Implications of all the available evidence**

There are at present no antenatal biomarkers that accurately predict GA over a wide window of pregnancy. Postnatal biomarkers appear more promising but are not available to guide antenatal care (such as who should receive magnesium sulphate for neuroprotection or steroids for lung maturation). Although metabolomic profiling from newborn blood spot appears more accurate than from cord blood, acceptability must also be considered. Further studies are needed in biomarker discovery; and to compare the most promising of these biomarkers in terms of accuracy and cost as well as equipment and infrastructure required. These are important considerations as biomarkers are most likely to be beneficial in LMIC settings, where these are significant barriers to implementation.

## Introduction

Accurate gestational age (GA) poses a significant global challenge, especially in Low- and Middle- Income Countries (LMICs). Recent research indicates that only 64 of 195 countries worldwide have national routine data to estimate preterm birth, meaning that estimates are heavily influenced by the lack of GA estimation.^1^ Relying on the reported last menstrual period (LMP) is often unreliable, due to inaccurate recall of dates, or irregular menstrual cycles, which is more common in breastfeeding women, those with polycystic ovarian syndrome or malnutrition.^2^ Although first trimester ultrasound scans are considered to be the gold standard for dating pregnancies,^3^^,^^4^ they are not commonly available in these settings at present, due to women presenting late for antenatal care.^5^ Later pregnancy dating using ultrasound biometry is much less accurate,^6^ and although novel tools have reduced this inaccuracy^7^^,^^8^ higher rates of inaccurate GA assessment remain an issue in the highest burden settings.

Accurate estimation of GA is crucial in obstetrics for multiple reasons. Firstly, at the level of the individual woman, it is essential for making obstetric decisions such as identifying patients who would benefit from interventions like steroids for fetal lung maturation^9^ or magnesium sulphate for neuroprotection^10^; interpreting diagnostic information such as malpresentation or a low lying placenta, which are only relevant near term. Secondly, at the level of the individual neonate, knowledge of GA is crucial to distinguish different types of small vulnerable newborns, i.e. babies that are small due to preterm birth or small for GA (SGA), ensuring they can receive appropriate care. Lastly, at the population level, knowledge of GA is essential to understand the prevalence of preterm birth as it is the leading cause of mortality in children under five years of age globally.^11^ This knowledge allows for targeted allocation of resources to improve outcomes.

Although estimating GA at birth through neonatal assessment is possible, this information is not available for prenatal care; and is often highly imprecise with estimates deviating by ±3 to 4 weeks from the gold standard.^12^ One way in which the assessment of GA may be improved is the use of biomarkers, such as those in maternal serum and urine, umbilical cord blood and neonatal heel prick testing. There would be an obvious benefit to an accurate, reliable and cost-effective biomarker that could estimate GA in this way. Therefore, our research question is what maternal or newborn biomarkers that assess GA exist. Several candidate biomarkers have been investigated, but to date no systematic evaluation of the accuracy of these biomarkers to estimate GA has been undertaken. This systematic review and meta-analysis aimed to identify, and determine the accuracy of, prenatal and postnatal biomarkers that have been proposed for the estimation of GA.

## Methods

This systematic review was prospectively registered with PROSPERO International Prospective Register of Systematic Reviews (PROSPERO identifier CRD42020167727) and reported in accordance with the Preferred Reporting Items for a Systematic Review and Meta-analysis of Diagnostic Test Accuracy Studies (PRISMA-DTA) statement.^13^

### Literature search

To identify potentially eligible studies, we searched MEDLINE(OvidSP)[1946-present], EMBASE (OvidSP)[1974-present], CINAHL (EBSCOHost)[1982-present], LILACS https://www.globalindexmedicus.net/, medRxiv https://www.medrxiv.org/, and Science Citation Index (Web of Science Core Collection)[1900-present] from inception to 15th September 2023. The search included a combination of subject headings and textwords for “*gestational age*”, “*estimation*” and “*biomarkers*” (Appendix S1). There were no language or date restrictions. In addition, we searched Google Scholar and screened proceedings of relevant conferences and reference lists of identified studies and relevant reviews.

### Eligibility criteria

Studies were included if they met the following criteria: (1) cohort or cross-sectional studies that assessed the accuracy of biomarkers in women with healthy singleton pregnancies or in any newborns for estimating GA at any point during pregnancy or at birth, respectively; (2) the gold standard GA age used in the study was based on the best obstetrical estimate (last menstrual period, dating ultrasound or a combination of both); and (3) the study reported at least one statistic assessing correlation or agreement of GA estimation, or diagnostic accuracy.

Studies were excluded if: (1) they were case–control studies, case series or reports, editorials, comments, or reviews without original data; (2) the gold standard GA used in the study was based on neonatal physical and neurologic assessment or was not reported; (3) they did not report any statistics assessing correlation or agreement of GA estimation or diagnostic accuracy, or sufficient information to calculate them could not be retrieved; (4) they included pregnancies or newborns with specific pathologies. In cases of duplicate publication, we selected the most recent and complete versions and supplemented if additional information appeared in the other publications.

### Assessment of risk of bias

The risk of bias in each included study was assessed independently by two authors (EB and AC-A) using a modified version of the QUADAS (Quality Assessment of Diagnostic Accuracy Studies)-2 tool.^14^ Disagreements in risk of bias assessment were resolved through consensus. We evaluated five domains believed to be important for the quality of studies evaluating the diagnostic accuracy of biomarkers for estimating GA. Each domain was scored as “low risk”, “high risk”, or “unclear risk” of bias. The domains evaluated and their interpretation, were as follows:

1. *Study design*—“low risk of bias”: pregnant women or newborns consecutively or randomly selected and prospective cohort design; “high risk of bias”: convenience sampling (arbitrary or non-consecutive recruitment) or retrospective cohort design.
2. *Description of the biomarker*—“low risk of bias”: the study report included a detailed description of the biomarker(s) assessed including sampling site, assay used, manufacturer of assay, GA at which the sample was collected (for prenatal biomarkers) and age at testing in hours after birth (for postnatal biomarkers), and frequency of testing; “high risk of bias”: if this information was not reported.
3. *Reference standard*—“low risk of bias”: GA that was established by early ultrasound measurement of fetal crown-rump length (between 8^+0^ weeks and 13^+6^ weeks), or by the woman’s LMP corroborated by early ultrasound, or by the woman’s LMP that was in agreement (within 7 days) with ultrasound measurements performed later in the pregnancy, or by certain ovulation date. This last criterion was not included in the PROSPERO protocol but the reviewers subsequently agreed it was appropriate to consider a study as a low-risk of bias for this domain; “high risk of bias”: GA that was not established according to the previously mentioned parameters.
4. *Blinding*—“low risk of bias”: GA based on biomarker(s) results was estimated without knowledge of the results of the “gold standard” GA; “high risk of bias”: GA based on biomarker(s) results was estimated with knowledge of the results of the “gold standard” GA. Our predefined protocol stated that an explicit statement that researchers were blind to the actual GA would render the method at low risk of bias. Nevertheless, upon review of studies we concluded that for very large studies, it would be very unlikely that researchers would estimate GA based on knowledge the “gold standard” estimate of GA, and we included this criterion retrospectively as low risk of bias.
5. *Inclusion of participants in the primary analysis*—“low risk of bias”: if at least 90% of enrolled women/newborns in the study were included in the primary analysis; “high risk of bias”: if less than 90% of enrolled women/newborns in the study were included in the primary analysis.

If there was insufficient information available to make a judgment about the bias of a domain, then it was scored as “unclear risk of bias”.

### Data extraction

Two reviewers (EB and AC-A) independently extracted data from each eligible study using a standardized data collection form. We resolved any disagreements by discussion and consensus. Information was extracted on study characteristics (first author’s name, date of publication, geographic location of the study, study design, recruitment of participants, time period for recruitment of participants, prospective or retrospective data collection, blinding, and completeness of follow up and reporting of withdrawals); participants characteristics (inclusion and exclusion criteria, sample size, and demographic characteristics); description of the biomarker(s) assessed (GA at sampling for prenatal biomarkers, age at testing in hours after birth for postnatal biomarkers, sampling site, frequency of test, analytical method used, cut-off values, biomarkers included in predictive models, and costs); reference standard used (definition of “gold standard” GA); outcomes (definition of outcomes); main findings of the study; and measures of diagnostic accuracy of biomarker(s) for estimating GA for the entire cohort and/or model development and validation subsets, and subgroups of participants (coefficient of correlation, coefficient of determination, standard error of estimation, root mean square error [RMSE], mean absolute error, proportion of mothers/infants with predicted GA within 1 and 2 weeks of GA estimated by the gold standard method, and area under receiver operating characteristic curve (AUC) with 95% confidence interval (CI) to discriminate GA across a dichotomous preterm birth threshold).

### Data synthesis

Studies that assessed prenatal biomarkers were grouped according to GA at which the biomarker was measured (4–10 weeks, 4–16 weeks, and all trimesters of pregnancy), whereas postnatal biomarkers were grouped according to sample site (neonate heel prick blood and cord blood). Meta-analyses were performed if at least two studies assessed the same biomarker(s) and reported similar measures of diagnostic accuracy for estimating GA. Data were synthesized in several ways.

First, we estimated pooled coefficients of correlation with 95% confidence intervals (CIs) for prenatal biomarkers (human chorionic gonadotrophin, pregnancy-specific beta-1-glycoprotein, human placental lactogen, and metabolomic profile in maternal serum) according to GA at which the biomarker was measured, and for postnatal biomarkers (DNA methylation profile in cord blood) by using the Hedges-Olkin (a conventional summary meta-analysis with a Fisher Z transformation of the correlation coefficient)^15^ and Hunter-Schmidt (a weighted mean of the raw correlation coefficient)^16^ methods. In this systematic review, the correlation coefficient measures the strength of the linear relationship between the GA predicted by the biomarker and that estimated by the gold standard method. It varies between −1 and 1 with 0 indicating no linear relationship, +1 indicating a perfect positive linear relationship, and −1 indicating a perfect negative linear relationship.

Second, pooled RMSE (overall average deviation in weeks between the GA predicted/estimated by the biomarkers and that estimated by the gold standard method) with 95% CI was estimated for metabolomic profile in maternal serum, metabolomic profile derived from newborn blood spot screening. This was done for all infants, and for subgroups of infants with a GA ≥37 weeks and <37 weeks, and infants born SGA (birthweight below the 10th percentile for GA). DNA methylation profile in cord blood from RMSEs, SDs and sample sizes reported in each study.

Third, pooled proportions of infants with predicted GA within 1 and 2 weeks of GA estimated by the gold standard method were calculated for metabolomic profile derived from newborn blood spot screening (heel prick blood and cord blood). Percentages (95% CIs) in individual studies were logit transformed to obtain pooled proportions with 95% CIs. Pooled proportions were obtained for all infants and for subgroups of infants with a GA ≥37 weeks, <37 weeks, 32–36 weeks, and <32 weeks, and infants born SGA (birthweight below the 10th percentile for GA). Finally, the method described by Zhou et al.^17^ was used to calculate the pooled AUC with 95% CI for metabolomic profile derived from newborn blood spot screening to discriminate infants with a GA <34 weeks from those with a GA ≥34 weeks, and infants with a GA <37 weeks from those with a GA ≥37 weeks.

All meta-analyses were performed using a random-effects model because we anticipated that there would be substantial heterogeneity between the results of the studies. The random effects model tends to give a more conservative estimate with wider 95% CI. Heterogeneity of the results among studies was evaluated by estimating the quantity *І*^*2*,^.^18^ A significant level of heterogeneity was defined as *І*^*2*^ ≥ 30%.^18^ We planned to explore potential sources of heterogeneity by performing subgroup and sensitivity analyses and to assess publication and related biases by examining the symmetry of funnel plots using Deeks’ test^19^; however, most of these analyses were not performed given the small number of studies included in most meta-analyses performed.

Statistical analyses were performed by using StatsDirect (Version 3.3.5; StatsDirect Ltd, Merseyside, United Kingdom).

### Ethics

As a systematic review, our study did not involve direct participation of human subjects and focused solely on previously published and publicly available data. It did not require institutional review board approval for this reason. The ethical principles governing this study adhere to the established guidelines for systematic reviews and meta-analyses and was based on registration of the protocol, search and analysis strategy to enhance transparency and preclude selective reporting.

### Role of the funding source

The funders of this study had no role in study design, data collection, analysis or interpretation of the data, in writing the paper or the decision to submit for publication.

## Results

### Selection, characteristics, and risk of bias of studies

**Fig. 1:**
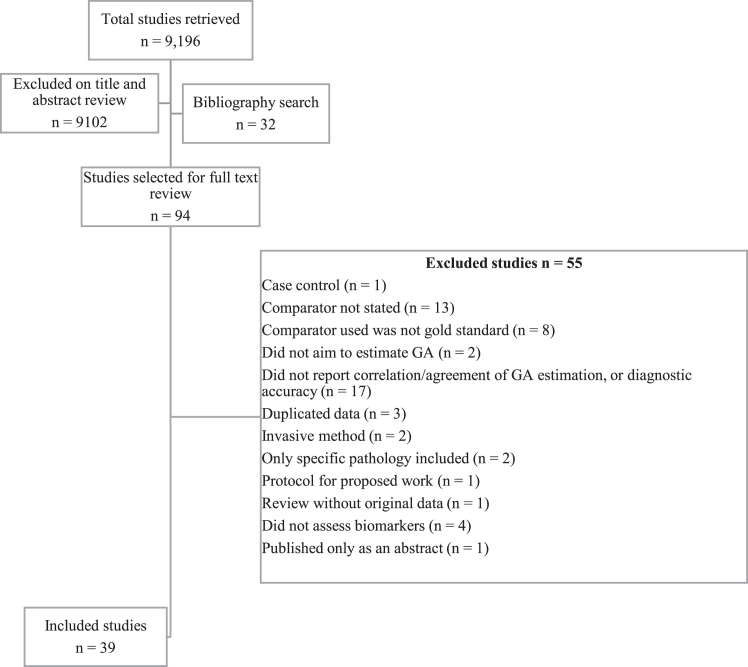
Flow diagram showing screening process of studies in biomarkers review; adapted from PRISMA.^20^

Our search returned 9,196 studies, of which 94 were selected for full text review. Of these, 55 were excluded based on the prespecified exclusion criteria discussed in Methods section (Fig. 1). References for excluded studies can be obtained from the authors upon request. The remaining 39 studies fulfilled the inclusion criteria.^21^, ^22^, ^23^, ^24^, ^25^, ^26^, ^27^, ^28^, ^29^, ^30^, ^31^, ^32^, ^33^, ^34^, ^35^, ^36^, ^37^, ^38^, ^39^, ^40^, ^41^, ^42^, ^43^, ^44^, ^45^, ^46^, ^47^, ^48^, ^49^, ^50^, ^51^, ^52^, ^53^, ^54^, ^55^, ^56^ Twenty studies, including 2050 women, assessed prenatal biomarkers,^21^, ^22^, ^23^, ^24^, ^25^, ^26^, ^27^, ^28^, ^29^, ^30^, ^31^, ^32^, ^33^, ^34^, ^35^, ^36^, ^37^, ^38^, ^39^, ^40^ and 19, including 1,738,652 newborns, assessed postnatal biomarkers.^41^, ^42^, ^43^, ^44^, ^45^, ^46^, ^47^, ^48^, ^49^, ^50^, ^51^, ^52^, ^53^, ^54^, ^55^, ^56^, ^57^, ^58^, ^59^

The main characteristics of the studies included in the systematic review are presented in Table 1, Table 2. Among studies that assessed prenatal biomarkers, 14 evaluated placental hormones (human chorionic gonadotrophin [hCG], human placental lactogen [hPL], pregnancy-specific beta-1-glycoprotein [SP1] and placental protein-14),^21^, ^22^, ^23^, ^24^, ^25^, ^26^, ^27^, ^28^, ^29^, ^30^, ^31^, ^32^, ^33^, ^34^ three evaluated metabolomic profiles,^37^^,^^38^^,^^40^ and one each evaluated plasma proteomics,^36^ cell-free RNA [cfRNA] transcripts,^35^ and exon-level gene expression^39^ (Table 1). Most studies (95%) were conducted in high-income countries (United States, United Kingdom, and Denmark). Only one study was conducted in LMICs. Among studies that assessed postnatal biomarkers, 13 evaluated metabolomic profiles (Acyl-carnitine, amino acid, fatty acid, ceramide, ceramide 1-phosphate, galactosylceramide, phosphatidyl acid, phosphatidylethanolamine, phosphatidylglycerol, phosphatidylinositol, phosphatidylcholine, cholesteryl ester, and sphingomyelin),^42^, ^43^, ^44^^,^^47^, ^48^, ^49^^,^^51^, ^52^, ^53^^,^^55^, ^56^, ^57^, ^58^ five evaluated genome methylation profiles (DNA methylation profiles),^45^^,^^46^^,^^50^^,^^54^^,^^59^ and one evaluated fetal haematological components (isoenzymes of erythrocytic carbonic anhydrase)^41^ (Table 2). Twelve (63%) studies were conducted in high-income countries and seven (37%) in LMICs.

**Table 1:** Characteristics and main findings of included studies that assessed prenatal biomarkers for predicting gestational age.

First author, year	Country (Region)	Design	Sample size	Biomarker(s)	Biological sample	Gestational age at testing	Reference standard	Main findings
Peeters, 1976^21^	USA (Colorado)	Prospective cohort	9	hCG hPL	Serum	10–25 weeks	GA estimated by LMP	At 12–17 weeks of gestation, hCG combined with hPL provided an estimate of GA within ±9.4 days of that provided by LMP; at 12–15 weeks of gestation, hPL alone provided an estimate of GA within ±12.3 days of that provided by LMP.
Lagrew, 1983^22^	USA (Kentucky)	Unclear	95	hCG	Serum	4–18.6 weeks	GA estimated by LMP	(1) The coefficients of determination (R^2^) between hCG and GA were 0.183, 0.826, and 0.326 from 4 to 18.6 weeks, <8.6 weeks, and ≥8.6 weeks, respectively. The mean difference (SD) between the GA predicted by β-hCG before 8.6 weeks and that predicted by LMP was 3.1 (1.8) days.
Whittaker, 1983^23^	UK (Newcastle)	Prospective cohort	35	hCG hPL	Serum	3–20 weeks	GA estimated by LMP	(1) The coefficient of correlation (*r*) between GA predicted by hCG and that estimated by LMP was 0.940 up to 8.6 weeks of gestation, with a SD of ±4.2 days. (2) The coefficient of correlation (*r*) between GA predicted by hPL and that estimated by LMP was 0.877 between 6 and 14 weeks of gestation with a SD of ±6.3 days.
Ahmed, 1984^24^	UK (Aberdeen)	Prospective cohort	34	hCG SP1	Serum	2–16.7 weeks	GA estimated by LMP	(1) The coefficients of correlation (*r*) between GA predicted by hCG and that estimated by LMP were 0.471 between 4.7 and 16.7 weeks of gestation, 0.796 between 5 and 10 weeks of gestation, and 0.833 between 3.4 and 9 weeks of gestation. The mean difference (SD) between GA predicted by hCG and that estimated by LMP was 4.4 (2.8) days between 3.5 and 9 weeks, and 12.0 (9.9) days between 3.5 and 16 weeks. (2) The coefficients of correlation (*r*) between GA predicted by SP1 and that estimated by LMP were 0.944 between 4.7 and 16.7 weeks of gestation and 0.910 between 5 and 10 weeks of gestation. The mean difference (SD) between GA predicted by SP1 and that estimated by LMP was 4.8 (4.3) days between 3.5 and 16 weeks.
Lagrew, 1984^25^	USA (Kentucky)	Prospective cohort	15	hCG	Serum	4.1–8.6 weeks	GA estimated by LMP	(1) The coefficient of correlation (*r*) between GA predicted by hCG and that estimated by LMP was 0.94. The mean difference (SD) between GA predicted by hCG and that estimated by LMP was 3.2 (2.5) days, with a range of 0.1–7.7 days.
Westergaard, 1985^26^	Denmark (Odense)	Prospective cohort	26	hCG SP1	Serum	4.3–8.6 weeks	GA estimated by LMP	(1) The coefficients of correlation (*r*) between GA predicted by hCG and SP1, and that estimated by LMP were 0.792 and 0.782, respectively. (2) The mean difference between GA predicted by hCG and SP1 and that estimated by LMP was 15.3 and 14.3 days, respectively.
Ahmed, 1985^27^	UK (Aberdeen)	Prospective cohort	56	hCG SP1	Serum	<5 (N = 13) and 5–16 (N = 43) weeks	GA estimated by LMP and ultrasound	(1) The coefficients of correlation (*r*) between GA predicted by hCG and that estimated by LMP/ultrasound were 0.727 at or before 5 weeks of gestation, 0.834 between 5 and 9 weeks of gestation, and 0.401 between 5 and 16 weeks of gestation. (2) The coefficients of correlation (*r*) between GA predicted by SP1 and that estimated by LMP/ultrasound were 0.672 (at or before 5 weeks of gestation), 0.917 between 5 and 9 weeks of gestation, and 0.952 between 5 and 16 weeks of gestation.
Ahmed, 1986^28^	UK (Aberdeen)	Prospective cohort	62	SP1	Serum	5–16 weeks	GA estimated by LMP	When GA predicted by SP1 was greater than that estimated by LMP, the mean difference (SD) was 6.5 (5.8) days. When GA predicted by SP1 was lower than that estimated by LMP, the mean difference (SD) was 2.6 (1.4) days.
Bersinger, 1986^29^	UK (Aberdeen)	Prospective cohort	139	SP1	Serum and urine	4–16 weeks	GA estimated by LMP	(1) The coefficients of determination (R^2^) between SP1 in serum and GA were 0.60, 0.52, 0.73, 0.78, 0.80, and 0.77 at 6, 8, 10, 12, 14, and 16 weeks, respectively. (1) The coefficients of determination (R^2^) between SP1 in urine and GA were 0.05, 0.25, 0.57, 0.66, 0.70, and 0.71 at 6, 8, 10, 12, 14, and 16 weeks, respectively.
Chervenak, 1986^30^	USA (New York)	Retrospective cohort	77	hCG	Serum	4.0–8.6 weeks	GA estimated by ovulation date	The average prediction error (gestational age estimated by ovulation date minus gestational age predicted by hCG) varied between 2.1 days (SD, 3.6) and 4.1 days (SD, 6.8)
Whittaker, 1987^31^	UK (Newcastle)	Prospective cohort	585	hPL	Serum	6.3–18.9 weeks	GA estimated by LMP	(1) The coefficient of correlation (*r*) between GA predicted by hPL and that estimated by LMP was 0.781. (2) The mean difference (SD) between GA predicted by hPL and that estimated by LMP was 0.3 (8.4) days. (3) Compared with LMP, hPL dated 91% of pregnancies within ±13 days.
Thomson, 1988^32^	UK (Aberdeen)	Prospective cohort	233	• SP1 • hPL	Serum	<16 weeks	GA estimated by LMP	(1) The coefficients of correlation (*r*) between GA predicted by SP1 and hPL and that estimated by LMP were 0.337 and 0.696, respectively. (2) The mean difference (SD) between GA predicted by SP1 and hPL, and that estimated by LMP was 2.2 (22.7) days and 5.2 (11.7) days, respectively.
Johal, 1991^33^	UK (London)	Prospective cohort	100	• hCG • hPL • PP-14	Serum	5.7–11.6 weeks	GA estimated by LMP and ultrasound	(1) The coefficients of correlation (*r*) between serum levels of hCG, hPL and PP-14, and GA estimated by LMP/ultrasound were −0.062, 0.599, and 0.02, respectively. (2) The accuracy of hPL in dating a pregnancy was ±16 days, as compared with dating by ultrasound.
Larsen, 2013^34^	USA (multicity)	Prospective cohort	178	hCG	Urine	0–8 weeks	GA estimated by ovulation date, LMP and ultrasound	The agreement between the GA based on the hCG concentration and that based on the ovulation day was 95.9% for a GA of 1–2 weeks, 93.4% for 2–3 weeks, and 95.2% for 3–8 weeks.
Ngo, 2018^35^	Denmark (Copenhagen) and USA (Pennsylvania and Birmingham)	Prospective cohort	31 full-term pregnancies (Denmark) and 38 pregnancies at risk for preterm birth (USA)	Cell-free RNA transcripts	Serum	Second and third trimester	GA estimated by ultrasound	(1) A model of 51 cell-free RNA transcripts predicted GA with a correlation coefficient of 0.91 and 0.89 in discovery and validation cohorts, respectively. (2) Among term pregnancies, estimates of GA predicted by a model of cell-free RNA transcripts measured at both second and third trimesters fell within ±7 days of the observed GA at delivery with an accuracy of 45%, as compared to 48% for estimates of ultrasound at first trimester. (3) The AUC of cell-free RNA in differentiating spontaneous preterm (<37 weeks) and term (≥37 weeks) deliveries was 0.86 and 0.81 in discovery and validation cohorts, respectively.
Aghaeepour, 2018^36^	USA (Stanford)	Prospective cohort	27 (17 in training cohort and 10 in validation cohort)	Proteomic profile	Plasma	7-14, 15–20, and 24–32 weeks	GA estimated by LMP and ultrasound	(1) A model including 74 proteins predicted gestational age with a correlation coefficient of 0.97 and 0.94 in the training and validation cohorts, respectively. (2) A model including 8 proteins predicted gestational age with a correlation coefficient of 0.97 and 0.91 in the training and validation cohorts, respectively.
Sylvester, 2020^37^	USA (Stanford and Birmingham)	Retrospective cohort	58 (36 in model development cohort and 22 in the validation cohort)	Metabolomic profile^a^	Serum	First, second and third trimester	GA estimated by ultrasound	(1) A model including 13 categories of metabolites predicted gestational age with a coefficient of determination (R^2^) of 0.98 and 0.81 in the development and validation cohorts, respectively. (2) The average difference between GA predicted by the model of metabolomic profile and that estimated by ultrasound was 1.09 weeks in the development cohort and 2.36 weeks in the validation cohort. (3) The model predicted GA within 1 week of GA estimated by ultrasound for 66.7% of women and within 2 weeks for 77.8% of women. (4) The AUC of a second model (using a set of 10 metabolic pathways) to discriminate gestational age <35 versus ≥37 weeks was 0.96 and 0.92 in the development and validation cohorts, respectively.
Liang, 2020^38^	Denmark	Prospective cohort	38 (21 in discovery cohort, 9 in a first validation cohort, and 8 in a second validation cohort)	Metabolomic profile^b^	Plasma	Weekly, from 5 weeks to postpartum period	GA estimated by ultrasound	(1) A model of 42 metabolites predicted GA with correlation coefficients of 0.96 and 0.95 in discovery and validation cohorts, respectively, and RMSEs of 2.49 and 2.76 weeks in discovery and validation cohorts, respectively. (2) A model of 5 metabolites (THDOC, estriol-16-glucuronide, progesterone, PE (P-16:0e/0:0), and DHEA-S) predicted GA with correlation coefficients of 0.92, 0.89, and 0.91 in discovery, validation cohort 1, and validation cohort 2, respectively, and RMSEs of 3.67, 4.11, and 3.05 weeks in discovery, validation cohort 1, and validation cohort 2, respectively. (3) The AUC of a model of 3 metabolites (THDOC, estriol-16-glucuronide, and androstane-3,17-diol) to discriminate gestational age <37 versus ≥37 weeks was 0.91 and 0.87 in the discovery and validation cohort 1, respectively.
Tarca, 2021^39^	United States	Prospective cohort	133	Exon-level gene expression	Whole blood	8 to >37 weeks	GA estimated by ultrasound	(1) Whole-blood gene expression predicted gestational age in both normal and complicated pregnancies with a correlation coefficient of 0.83 and a RMSE of 4.5 weeks.
Contrepois, 2022^40^	Bangladesh, Pakistan, Tanzania, and Zambia (discovery cohort); United States (validation cohort)	Retrospective cohort	119 (99 in discovery cohort and 20 in validation cohort)	Metabolomic profile^c^	Urine	8–19 weeks	GA estimated by LMP and ultrasound	(1) A model of 3 metabolites predicted GA with correlation coefficients of 0.87 and 0.70 in discovery and validation cohorts, respectively, and RMSEs of 1.58 and 2.40 weeks in discovery and validation cohorts, respectively. (2) Gestational age was predicted more accurately among women with term deliveries (correlation coefficient and RMSE of 0.89 and 1.34 weeks, respectively) than among women with preterm deliveries (correlation coefficient and RMSE of 0.69 and 2.32 weeks, respectively)

AUC, area under the curve; CRL, crown-rump length; DHEA-S, dehydroepiandrosterone-sulfate; GA, gestational age; hCG, human chorionic gonadotrophin; hPL, human placental lactogen; LMP, last menstrual period; PE(P-16:0e/0:0), 1-(1Z-hexadecenyl)-sn-glycero-3-phosphoethanolamine; PP, placental protein; RMSE, root mean squared error; SD, standard deviation; SP1, pregnancy-specific beta-1-glycoprotein; THDOC, tetrahydrodeoxycorticosterone.

a Acyl-carnitine, amino acid, fatty acid, ceramide, ceramide 1-phosphate, galactosylceramide, phosphatidyl acid, phosphatidylethanolamine, phosphatidylglycerol, phosphatidylinositol, phosphatidylcholine, cholesteryl ester, and sphingomyelin.

b THDOC, Estriol-16-Glucoronide, Progesterone, PE (P-16:Oe/0:0) and DHEA-S.

c C19H26O7S, C24H30O9 and estriol glucuronide.

**Table 2:** Characteristics and main findings of included studies that assessed postnatal biomarkers for predicting gestational age.

First author, year	Country (Region)	Design	Sample size	Biomarker(s)	Biological sample	Newborn age at testing	Reference standard	Main findings
Moynihan, 1977^41^	Ireland (Dublin)	Prospective cohort	45	Isoenzymes A, B and C of erythrocytic carbonic anhydrase	Cord blood	At birth	GA estimated by LMP	(1) The coefficient of correlation (*r*) between the ratio of activity of isoenzyme B to activity of isoenzyme C of erythrocytic carbonic anhydrase and GA between 35 and 41.5 weeks was 0.94. The coefficient of correlation (*r*) between total activity of erythrocytic carbonic anhydrase and GA was 0.69.
Wilson, 2016^42^	Canada (Ontario, April 2007–March 2009)	Retrospective cohort	249,700 (124,854 in model development dataset; 62,412 in validation dataset; and 62,434 in test dataset)	Metabolomic profile derived from newborn blood spot screening^a^	Heel prick blood	24–72 h	GA estimated by LMP and/or ultrasound	(1) The average difference between GA predicted by the full model of metabolomic profile (44 markers), birthweight and sex, and that estimated by LMP/ultrasound was 1.06 weeks. (2) The prediction of GA by the full model was more accurate for term infants (RMSE, 0.97 weeks) and non-SGA infants (RMSE, 1.03 weeks) than for preterm infants (RMSE between 1.70 and 2.30 weeks) and SGA infants (RMSE, 1.34 weeks). (3) The full model predicted GA within 1 week of GA estimated by LMP/ultrasound for 66.8% of infants and within 2 weeks for 94.9% of infants. (4) The AUC of the full model in differentiating infants born before 37 weeks from those born at or after 37 weeks was 0.970 (95% CI, 0.966–0.974). (5) The AUC of the full model in differentiating infants born before 34 weeks from those born at or after 34 weeks was 0.991 (95% CI, 0.987–0.995).
Ryckman, 2016^43^	USA (Iowa)	Retrospective cohort	230,013 (153,342 in model-building dataset; 76,671 in model-testing dataset)	Metabolomic profile derived from newborn blood spot screening^b^	Heel prick blood	0–24 h (14%); 25–72 h (84%); >72 h (2%)	GA estimated by LMP and/or ultrasound	(1) The average difference between GA predicted by the metabolomic model and that estimated by LMP/ultrasound was 1.3 weeks. The inclusion of neonatal weight in the metabolomic model reduced the average difference to 1.1 weeks. (2) The average difference between GA predicted by the metabolomic model and that estimated by LMP/ultrasound among infants born SGA and large-for GA was 1.5 and 1.4 weeks, respectively. (3) The metabolomic model predicted GA within 1 week of GA estimated by LMP/ultrasound for 78% of infants and within 2 weeks for 95% of infants. (4) The AUC of the metabolomic model in differentiating infants born before 37 weeks from those born at or after 37 weeks was 0.899 (95% CI, 0.895–0.903). The inclusion of neonatal weight in the metabolomic model increased the AUC to 0.938 (95% CI, 0.934–0.941).
Jelliffe-Pawlowski, 2016^44^	USA (California)	Retrospective cohort	729,503 (547,127 in training dataset; 182,376 in testing dataset)	Metabolomic profile derived from newborn blood spot screening^c^	Heel prick blood	12 h to 8 days (91% between 12 and 72 h)	GA estimated by ultrasound	(1) Among infants born before 37 weeks, the model of metabolomic profile (35 markers), birthweight, and hours of age at testing predicted GA within 1 week of GA estimated by ultrasound for 78.3% of infants and within 2 weeks for 91.7% of infants. (2) The model was more accurate to predict GA among infants born between 32 and 36 weeks (80.2% of infants within 1 week and 92.5% within 2 weeks of GA estimated by ultrasound) than among infants born before 32 weeks (53.1% of infants within 1 week and 73.7% within 2 weeks of GA estimated by ultrasound). (3) Overall, the model had a sensitivity of 99.5% and a specificity of 98.9% to sort preterm and term births accurately. Sensitivities and specificities were ≥94.9% in the subgroups of SGA, appropriate-for-GA, and large-for-GA infants.
Knight, 2016^45^	Multicountry	Retrospective cohort	1342 (207 in training dataset, and 1135 in testing dataset)	DNA methylation profile	Cord blood (872 neonates) and heel prick blood (470 neonates)	From birth up to 39 days	GA estimated by LMP and/or ultrasound	(1) The coefficients of correlation (*r*) between GA predicted by DNA methylation and that estimated by LMP/ultrasound were 0.99 and 0.91 in training and testing datasets, respectively. The average difference between GA predicted by DNA methylation and that estimated by LMP/ultrasound was 1.49 weeks (SD, 1.16 weeks). (2) The coefficients of correlation (*r*) between GA predicted by DNA methylation and that estimated by LMP/ultrasound were 0.57 and 0.95 in cord blood and heel prick blood samples, respectively. (3) GA predicted by DNA methylation correlated more strongly with GA estimated by ultrasound (*r* = 0.54) than that estimated exclusively by LMP (*r* = 0.41).
Bohlin, 2016^46^	Norway	Retrospective cohort	1753 (1068 in training dataset, and 685 in replication dataset)	DNA methylation profile	Cord blood	At birth	GA estimated by LMP and ultrasound	(1) DNA methylation had a coefficient of determination (R^2^) of 0.66 and provided an estimate of GA within ±1.79 weeks of that provided by ultrasound. (2) DNA methylation had a coefficient of determination (R^2^) of 0.50 and provided an estimate of GA within ±2.13 weeks of that provided by LMP.
Hawken, 2017^47^	Canada (Ontario, April 2009–September 2011)	Retrospective cohort	300,132 (validation cohort)	Metabolomic profile derived from newborn blood spot screening^a^	Heel prick blood	24 h to 7 days	GA estimated by LMP and/or ultrasound	(1) The average difference between GA predicted by the full model of metabolomic profile (44 markers), birthweight and sex, and that estimated by LMP/ultrasound was 1.04 weeks. The average difference was 1.05 weeks among infants born to non-immigrant mothers, and between 0.98 and 1.15 weeks among infants born to immigrant mothers. (2) The full model predicted GA within 1 week of GA estimated by LMP/ultrasound for 67.1% of infants and within 2 weeks for 95.0% of infants. (3) The AUC of the full model in differentiating infants born before 37 weeks from those born at or after 37 weeks was 0.957 (95% CI, 0.956–0.959). (4) The AUC of the full model in differentiating infants born before 34 weeks from those born at or after 34 weeks was 0.981 (95% CI, 0.979–0.983). (5) In the non-immigrant subgroup, the AUC of the full model to discriminate gestational age <37 versus ≥37 weeks and ≤34 versus >34 weeks was 0.958 and 0.986, respectively. (6) In the immigrant subgroups, the AUC of the model to discriminate gestational age <37 versus ≥37 weeks and ≤34 versus >34 weeks ranged from 0.927 to 0.964 and from 0.966 to 0.994, respectively.
Wilson, 2017^48^	Canada (Ontario, January 2012–December 2014)	Retrospective cohort	159,215 (79,620 in model development dataset; 39,785 in validation dataset; and 39,810 in test dataset)	Metabolomic profile derived from newborn blood spot screening^d^	Heel prick blood	<48 h	GA estimated by ultrasound	(1) The average difference between GA predicted by the full model of metabolomic profile (all markers including haemoglobin ratio), birthweight, sex, and multiple birth status, and that estimated by ultrasound was 1.04 weeks. (2) The prediction of gestational age was more accurate for term infants (RMSE, 1.01 weeks) and late preterm infants (RMSE, 1.16 weeks) than for preterm infants <34 weeks (RMSE, 2.24 weeks) and SGA infants (RMSE, 1.52 weeks). (3) The full model predicted GA within 1 week of GA estimated by ultrasound for 68.7% of infants and within 2 weeks for 95.3% of infants. (4) The AUC of the full model to discriminate gestational age <37 versus ≥37 weeks and ≤34 versus >34 weeks was 0.957 and 0.988, respectively. (5) Among SGA infants, the AUC of the model to discriminate gestational age <37 versus ≥37 weeks and ≤34 versus >34 weeks was 0.970 and 0.997, respectively.
Murphy, 2019^49^	Bangladesh	Prospective cohort	1069 (external validation cohort)	Metabolomic profile derived from newborn blood samples^e^	Cord blood (1036 samples) and heel prick blood (487 samples)	0 min to 2 h (cord blood); 25 min to 40 h (heel prick blood)	GA estimated by ultrasound	(1) The average difference between GA predicted by the full model of metabolomic profile (all markers), birthweight, sex, and multiple birth status, and that estimated by ultrasound was 1.07 weeks for heel prick blood samples and 1.23 weeks for cord blood samples. (2) The full model based on heel prick blood samples predicted GA within 1 week of GA estimated by ultrasound for 63.9% of infants and within 2 weeks for 94.3% of infants. (3) The full model based on cord blood samples predicted GA within 1 week of GA estimated by ultrasound for 59.4% of infants and within 2 weeks for 90.4% of infants. (4) Among SGA infants (below the 10th percentile for gestational age), the full model based on heel prick blood samples had an RMSE of 1.12 weeks, and predicted GA within 1 week of GA estimated by ultrasound for 62.8% of infants and within 2 weeks for 94.3% of infants. The full model based on cord blood samples had an RMSE of 1.20 weeks and predicted GA within 1 week of GA estimated by ultrasound for 63.1% of infants and within 2 weeks for 90.7% of infants. (5) Among LBW infants (<2500 g), the full model based on heel prick blood samples had an RMSE of 1.21 weeks and predicted GA within 1 week of GA estimated by ultrasound for 59.1% of infants and within 2 weeks for 94.3% of infants. The full model based on cord blood samples had an RMSE of 1.44 weeks and predicted GA within 1 week of GA estimated by ultrasound for 53.3% of infants and within 2 weeks for 84.2% of infants. (6) The AUC to discriminate gestational age <37 versus ≥37 weeks was 0.945 (95% CI, 0.890–0.999) for the full model based on heel prick blood samples, and 0.894 (95% CI, 0.853–0.935) for the full model based on cord blood samples.
Michaeli, 2019^50^	Israel (Jerusalem)	Prospective cohort	41 (10 in training group and 31 in test group)	DNA methylation profile	Cord blood and placental samples	At birth	GA estimated by LMP and ultrasound	(1) The coefficient of correlation (*r*) between GA predicted by DNA methylation (combination of demethylation and de novo methylation) and that estimated by LMP and ultrasound was 0.77 for cord blood samples. (2) The coefficient of correlation (*r*) between GA predicted by DNA methylation (demethylation alone) and that estimated by LMP and ultrasound was −0.55 for placental samples.
Hawken, 2020^51^	Zambia (Lusaka) and Bangladesh (Matlab)	Prospective cohort	1487 (external validation cohort)	Metabolomic profile derived from newborn blood samples^f^	Heel prick blood (662 infants) and cord blood (1404 infants)	At birth (cord blood); 24–72 h (heel prick blood)	GA estimated by ultrasound	(1) The full model based on heel prick blood samples had a mean absolute error of 0.79 weeks and predicted GA within 1 week of GA estimated by ultrasound for 69.4% of infants and within 2 weeks for 97.6% of infants in Zambia (2) The full model based on cord blood samples had a mean absolute error of 1.02 weeks and predicted GA within 1 week of GA estimated by ultrasound for 60.7% of infants and within 2 weeks for 90.1% of infants in Zambia. (3) The full model based on heel prick blood samples had a mean absolute error of 0.81 weeks and predicted GA within 1 week of GA estimated by ultrasound for 68.4% of infants and within 2 weeks for 94.7% of infants in Bangladesh. (4) The full model based on cord blood samples had a mean absolute error of 0.95 weeks and predicted GA within 1 week of GA estimated by ultrasound for 61.0% of infants and within 2 weeks for 91.2% of infants in Bangladesh.
Hawken, 2021^52^	China (Shanghai)	Retrospective cohort	4448 (external validation cohort)	Metabolomic profile derived from newborn blood spot screening^g^	Heel prick blood	<72 h	GA estimated by ultrasound	(1) The average difference between GA predicted by the full model of metabolomic profile (all markers), birthweight, and sex and that estimated by ultrasound was 1.20 weeks. The average deviation of the model estimate compared to the reference estimate (mean absolute error) was 0.89 weeks. (2) The full model predicted GA within 1 week of GA estimated by ultrasound for 64.7% of infants and within 2 weeks for 92.7% of infants. (3) Among preterm infants (<37 weeks), the full model had a mean absolute error of 1.74 weeks and an RMSE of 2.69 weeks, and predicted GA within 1 week of GA estimated by ultrasound for 43.4% of infants and within 2 weeks for 72.1% of infants. (4) Among SGA infants (below the 10th percentile for gestational age), the full model had a mean absolute error of 1.48 weeks and an RMSE of 1.70 weeks, and predicted GA within 1 week of GA estimated by ultrasound for 30.9% of infants and within 2 weeks for 77.3% of infants.
Oltman, 2021^53^	Uganda (Busia)	Prospective cohort	666 (external validation cohort)	Metabolomic profile derived from newborn blood samples^h^	Heel prick blood (666 infants) and cord blood (640 infants)	At birth (cord blood); ≤3 h (heel prick blood)	GA estimated by ultrasound	(1) The metabolomic model based on heel prick blood samples predicted GA within 1 week of GA estimated by ultrasound for 62.9% of infants and within 2 weeks for 89.2% of infants. (2) The metabolomic model based on cord blood samples predicted GA within 1 week of GA estimated by ultrasound for 60.9% of infants and within 2 weeks for 89.2% of infants. (3) Among SGA infants (Intergrowth definition), the metabolomic model based on heel prick blood samples predicted GA within 1 week of GA estimated by ultrasound for 44.3% of infants and within 2 weeks for 75.3% of infants. The metabolomic model based on cord blood samples predicted GA within 1 week of GA estimated by ultrasound for 41.5% of infants and within 2 weeks for 73.6% of infants. (4) The AUC to discriminate gestational age <37 versus ≥37 weeks was 0.953 (95% CI, 0.921–0.985) for the model of metabolomic profile based on heel prick blood samples plus birthweight, and 0.935 (95% CI, 0.894–0.977) for the model of metabolomic profile based on cord blood samples plus birthweight.
Haftorn, 2021^54^	Norway and Finland	Retrospective cohort	1941 (1429 in training dataset, and 512 in replication dataset)	DNA methylation profile	Cord blood	At birth	GA estimated by LMP and ultrasound	(1) DNA methylation had a coefficient of determination (R^2^) of 0.724 with a median absolute deviation of 3.42 days (and provided an estimate of GA within ±0.73 weeks of that provided by ultrasound) (2) Among ART-conceived newborns with known embryo transfer date, DNA methylation had a coefficient of determination (R^2^) of 0.767 with a median absolute deviation of 3.80 days (and provided an estimate of GA within ±0.76 weeks).
Sazawal, 2021^55^	Tanzania, Pakistan and Bangladesh	Retrospective cohort	1311	Metabolomic profile derived from newborn blood spot screening^i^	Heel prick blood	24–72 h	GA estimated by ultrasound	(1) The metabolomic model had an RMSE of 1.65 weeks and predicted GA within 1 week of GA estimated by ultrasound for 68.7% of infants and within 2 weeks for 88.6% of infants. (2) The inclusion of birthweight in the metabolomic model had an RMSE of 1.52 weeks and predicted GA within 1 week of GA estimated by ultrasound for 70.5% of infants and within 2 weeks for 90.1% of infants. (3) The AUC of the metabolomic and birthweight model in differentiating infants born before 37 weeks from those born at or after 37 weeks was 0.86 (95% CI, 0.83–0.89). (4) The model with metabolites and birthweight had a sensitivity of 80.7% and a specificity of 77.6% for differentiating between term and preterm infants.
Jasper, 2022^56^	Uganda	Retrospective cohort	150 (external validation cohort)	Metabolomic profile derived from cord blood samples^j^	Cord blood	At birth	GA estimated by ultrasound	(1) The model including metabolites and birthweight had an RMSE of 1.55 weeks and predicted GA within 2 weeks of GA estimated by ultrasound for 76.7% of infants. (2) The AUC of the metabolomic model in differentiating infants born before 37 weeks from those born at or after 37 weeks was 0.765 (95% CI, 0.596–0.935). The inclusion of birthweight in the metabolomic model increased the AUC to 0.851 (95% CI, 0.722–0.981). (3) The model with metabolites and birthweight had a sensitivity of 72.7% and a specificity of 82.7% for differentiating between term and preterm infants.
Hawken, 2022^57^	Canada (Ontario, January 2015–December 2017)	Retrospective cohort	52,659 (50,735 spontaneously conceived and 1924 conceived from ART)	Metabolomic profile derived from newborn blood spot screening^k^	Heel prick blood	<48 h	GA estimated by ultrasound and date of embryo transfer	(1) Overall, the model including metabolites and birthweight had a mean absolute error (95% CI) and an RMSE (95%) of 0.70 (0.69−0.70) weeks and 0.89 (0.88−0.90) weeks, respectively, and predicted GA within 1 week of GA estimated by ultrasound for 75.7% (95% CI, 75.4–76.1) of infants. (2) The prediction of GA was less accurate for preterm infants <37 weeks (mean absolute error, 0.91 [95% CI, 0.88−0.93] weeks; RMSE, 1.17 [95% CI, 1.13−1.20] weeks; predicted GA within 1 week of GA estimated by ultrasound for 62.6% [95% CI, 61.1−64.0] of infants) and SGA infants (mean absolute error, 1.15 [95% CI, 1.11−1.19] weeks; RMSE, 1.44 [95% CI, 1.38−1.50] weeks; predicted GA within 1 week of GA estimated by ultrasound for 49.1% [95% CI, 46.9−51.1] of infants). (3) When using GA estimated by date of embryo transfer as gold standard, the prediction of GA was slightly better for infants conceived from ART (mean absolute error, 0.68 [95% CI, 0.66−0.71] weeks; RMSE, 0.86 [95% CI, 0.83−0.88] weeks; predicted GA within 1 week of GA estimated by ultrasound for 75.4% [95% CI, 73.7−77.3] of infants).
Hawken, 2022^58^	Kenia	Prospective cohort	1039	Metabolomic profile derived from newborn blood samples^l^	Heel prick blood (1039 infants) and cord blood (1012 infants)	Within 30 min of delivery of the placenta (cord blood); 24–72 h (heel prick blood)	GA estimated by ultrasound	(1) The full model based on heel prick blood samples had a mean absolute error (95% CI) and an RMSE (95%) of 1.35 (1.27−1.43) weeks and 1.83 (1.72−1.94) weeks, respectively, and predicted GA within 1 week of GA estimated by ultrasound for 64.1% (95% CI, 61.1–67.2) of infants. (2) The full model based on heel prick blood samples was less accurate for preterm infants <37 weeks (mean absolute error, 2.62 [95% CI, 2.28−2.99] weeks; RMSE, 3.09 [95% CI, 2.74−3.48] weeks; predicted GA within 1 week of GA estimated by ultrasound for 29.4% [95% CI, 20.2−38.9] of infants) and SGA infants (mean absolute error, 1.81 [95% CI, 1.57−2.07] weeks; RMSE, 2.18 [95% CI, 1.91−2.46] weeks; predicted GA within 1 week of GA estimated by ultrasound for 46.9% [95% CI, 35.8−57.3] of infants). (3) The full model based on cord blood samples had a mean absolute error (95% CI) and an RMSE (95%) of 1.44 (1.36−1.53) weeks and 1.95 (1.85−2.06) weeks, respectively, and predicted GA within 1 week of GA estimated by ultrasound for 61.2% (95% CI, 58.3–63.9) of infants. (4) The full model based on cord blood samples was less accurate for preterm infants <37 weeks (mean absolute error, 2.79 [95% CI, 2.46−3.12] weeks; RMSE, 3.19 [95% CI, 2.85−3.57] weeks; predicted GA within 1 week of GA estimated by ultrasound for 21.1% [95% CI, 11.8−29.3] of infants) and SGA infants (mean absolute error, 2.06 [95% CI, 1.76−2.36] weeks; RMSE, 2.41 [95% CI, 2.13−2.69] weeks; predicted GA within 1 week of GA estimated by ultrasound for 33.2% [95% CI, 23.0−44.6] of infants).
Haftorn, 2023^59^	Norway	Retrospective cohort	2138 (1709 in training dataset, and 429 in replication dataset)	DNA methylation profile	Cord blood	At birth	GA estimated by LMP and ultrasound	(1) DNA methylation, including five CpGs, had a coefficient of determination (R^2^) of 0.674 with a median absolute deviation of 4.4 days

ART, assisted reproductive techniques; AUC, area under the curve; CI, confidence interval; GA, gestational age; LBW, low birth weight; LMP, last menstrual period; RMSE, root mean square error; SD, standard deviation; SGA, small for gestational age.

a Acyl-carnitines (C0, C2, C3, C4, C5, C6, C8, C8:1, C10, C10:1, C12, C12:1, C14, C14:1, C14:2, C16, C18, C18:1, C18:2), amino acids (arginine, phenylalanine, alanine, leucine, ornithine, citrulline, tyrosine, glycine, argininosuccinate, methionine, valine, biotinidine), fatty acid oxidation (C3DC, C4DC, C5OH, C5DC, C6DC), enzymes (galactose-1-phosphate uridyl transferase, biotinidase), and hormones (thyroid stimulating hormone, 17-hydroxyprogesterone).

b Acyl-carnitines (C0, C2, C3, C3-DC, C4, C4-DC, C5, C5:1, C5-DC, C5-OH, C6, C6-DC, C8, C8:1, C10, C10:1, C12, C12:1, C14, C14:1, C14:2, C14-OH, C16, C16:1, C16:1-OH, C16-OH, C18, C18:1, C18:1-OH, C18:2, C18:OH), amino acids (alanine, arginine, citrulline, glutamate, isoleucine + leucine, methionine, phenylalanine, tyrosine, valine), enzymes (galactose-1-phosphate uridyl transferase, biotinidase), and hormones (thyroid stimulating hormone, 17-hydroxyprogesterone).

c Free carnitine, acyl-carnitines (C-2, C-3, C-3DC, C-4, C-5, C-5:1, C-5DC, C-6, C-8, C-8:1, C-10, C-10:1, C-12, C-12:1, C-14, C-14:1, C-16, C-16:1, C-18, C-18:1, C-18:2, C-18:1OH), amino acids (alanine, arginine, citrulline, glycine, methionine, ornithine, phenylalanine, proline, 5-oxoproline, tyrosine, valine), thyroid stimulating hormone, 17-hydroxyprogesterone, and galactose-1-phosphate-uridyl-transferase.

d Acyl-carnitines (C0, C2, C3, C4, C5, C5:1, C6, C8, C8:1, C10, C10:1, C12, C12:1, C14, C14:1, C14:2, C16, C18, C18:1, C18:2, C10:1, C12:1, C14:1, C14:2, C4OH, C5:1, C5DC, C5OH, C6DC, C16:OH, C16:1OH, C18OH, C18:1OH, C3DC, C4DC), amino acids (alanine; arginine; citrulline; phenylalanine; leucine; ornithine; tyrosine; glycine; argininosuccinate; methionine; valine; succinylacetone), hemoglobins (adult haemoglobin: HbA(A) and variants (S, C, D, E) fetal haemoglobin: HbF (F), acetylated HbF (F1), combined HbF (F + F1)), endocrine markers (17α-hydroxyprogesterone (17-OHP), thyroid stimulating hormone (TSH)), and enzyme markers (biotinidase; galactose-1-phosphate uridyltransferase (GALT); immunotripsinogen).

e Acylcarnitines (N = 31), amino acids (N = 12), haemoglobin profiles, 17α-hydroxyprogesterone, thyroid stimulating hormone; immunoreactive trypsinogen, t-cell receptor excision circles, biotinidase activity, galactose-1-phosphate uridylyltransferase activity.

f Acyl-carnitines (C0; C2; C3; C4; C5; C5:1; C6; C8; C8:1; C10; C10:1; C12; C12:1; C14; C14:1; C14:2; C16; C18; C18:1; C18:2; C10:1; C12:1; C14:1; C14:2; C4OH; C5:1; C5DC; C5OH; C6DC; C16:OH; C16:1OH; C18OH; C18:1OH; C3DC; C4DC), amino acids (Arginine, phenylalanine, alanine, leucine, ornithine, citrulline, tyrosine, glycine, methionine, valine), hormones (thyroid stimulating hormone, 17-hydroxyprogesterone), haemoglobins (adult haemoglobin, fetal haemoglobin, and acetylated HbF), and enzyme markers (biotinidase and immunotripsinogen).

g Acyl-carnitines (C0, C2, C3, C4, C5, C6, C8, C10, C12, C14, C16, C18, C10:1, C12:1, C14OH, C14:1, C14:2, C16OH, C18OH, C18:1, C18:2, C3DC, C4DC, C4OH, C5DC, C5OH, C5:1, C6DC, C8:1), TSH, 17OHP, alanine, arginine, citruline, glycine, leucine, methionine, ornithine, phenylalanine, tyrosine, and valine.

h Acyl-carnitines (free carnitine, C2, C3, C4, C4-DC, C4-OH, C5, C5-OH, C6, C8, C10, C12, C12:1, C14, C14:1, C16, C16:1, C16:1-OH, C18, C18:1, C18:2), amino acids (alanine, arginine, citrulline, glutamate, leucine, methionine, ornithine, phenylalanine, succinylacetone, tyrosine, valine), and hormones (thyroid stimulating hormone, 17-hydroxyprogesterone).

i Alanine, arginine, isoleucine + leucine, methionine, phenylalanine, tyrosine, valine, C2, C3, C3-DC, C4, C4-DC, C5, C5:1, C5-OH, C5-DC, C6, C6-DC, C8, C8:1, C10, C10:1, C12, C12:1, C14, C14-OH, C16, C16:1, C16-OH, C16:1-OH, C18, C18:1, C18:1OH, C18:2, GALT, 17-hydroxyprogesterone, thyroid stimulating hormone.

j Arginosuccinate, isoleucine + leucine, methionine, phenylalanine, tyrosine, valine, C3, C4, C4-DC, C4-OH, C5, C8:1, C14, C14:1, C16, C18-OH, and C18:1.

k Haemoglobins (adult haemoglobin, fetal haemoglobin, and acetylated HbF), endocrine markers (17-hydroxyprogesterone, thyroid stimulating hormone), amino acids (arginine, phenylalanine, alanine, leucine, ornithine, citrulline, tyrosine, glycine, methionine, valine), acyl-carnitines (C0, C2, C3, C4, C5, C5:1, C6, C8, C8:1, C10, C10:1, C12, C12:1, C14, C14:1, C14:2, C16, C18, C18:1, C18:2, C10:1, C12:1, C14:1, C14:2, C4OH, C5:1, C5DC, C5OH, C6DC, C16:OH, C16:1OH, C18OH, C18:1OH, C3DC, C4DC), enzyme markers (biotinidase, immunoreactive trypsinogen), and immune markers (T-cell receptor excision circles).

l Haemoglobins (adult haemoglobin, fetal haemoglobin, and acetylated HbF), endocrine markers (17-hydroxyprogesterone, thyroid stimulating hormone), amino acids (arginine, phenylalanine, alanine, leucine, ornithine, citrulline, tyrosine, glycine, methionine, valine), acyl-carnitines (C0, C2, C3, C4, C5, C5:1, C6, C8, C8:1, C10, C10:1, C12, C12:1, C14, C14:1, C14:2, C16, C18, C18:1, C18:2, C10:1, C12:1, C14:1, C14:2, C4OH, C5:1, C5DC, C5OH, C6DC, C16:OH, C16:1OH, C18OH, C18:1OH, C3DC, C4DC), enzyme markers (biotinidase, immunoreactive trypsinogen, galactose-1-phosphate uridylyltransferase), and immune markers (T-cell receptor excision circles).

Biomarkers were obtained from the following biological samples: maternal serum^21^, ^22^, ^23^, ^24^, ^25^, ^26^, ^27^, ^28^, ^29^, ^30^, ^31^, ^32^, ^33^^,^^35^^,^^37^ (six biomarkers), maternal plasma^36^^,^^38^ (two biomarkers), maternal whole blood^39^ (one biomarker), maternal urine^29^^,^^34^ (two biomarkers), cord blood^41^^,^^45^^,^^46^^,^^49^, ^50^, ^51^^,^^53^^,^^54^^,^^56^^,^^58^^,^^59^ (three biomarkers), newborn blood spot^42^, ^43^, ^44^, ^45^^,^^47^, ^48^, ^49^^,^^51^, ^52^, ^53^^,^^55^^,^^57^^,^^58^ (2 biomarkers) and placental sample^50^ (one biomarker). Further breakdown of this is available in box 1. Biomarkers were evaluated throughout the three trimesters as well as in the immediate postnatal period: first trimester (18 studies), second trimester (nine studies), third trimester (five studies) and postnatally (21 studies). Samples were collected once only (22 studies), serially (12 studies) and frequency was not clearly defined in five studies.

Box 1 **Potential biomarkers identified and type of biological sample analysed.**

**Prenatal biomarkers**

1. Maternal serum

cfRNA

hCG

hPL

Sp1

Placental protein 14

Metabolomic profile (Acyl-carnitine, amino acid, fatty acid, ceramide, ceramide 1-phosphate, galactosylceramide, phosphatidyl acid, phosphatidylethanolamine, phosphatidylglycerol, phosphatidylinositol, phosphatidylcholine, cholesteryl ester, and sphingomyelin)

2. Maternal plasma

Proteomic profile

Metabolomic profile (THDOC, Estriol-16-Glucoronide, Progesterone, PE (P-16:Oe/0:0) and DHEA-S)

3. Maternal whole blood

Exon-level gene expression

4. Maternal urine

Sp1

Metabolomic profile (C19H26O7S, C24H30O9 and estriol glucuronide)

**Postnatal biomarkers**

1. Cord blood

DNA methylation profile

Isoenzymes of erythrocytic carbonic anhydrase (A, B and C)

Metabolomic profile (Acyl-carnitines, amino acids, enzymes, enzyme markers, fatty acid oxidation, free carnitine, haemoglobins (adult haemoglobin: HbA(A) and variants (S, C, D, E) fetal haemoglobin: HbF (F), acetylated HbF (F1), combined HbF (F + F1)), hormones (Thyroid stimulating hormone, 17-hydroxyprogesterone, galactose-1-phosphate-uridyl-transferase), immunoreactive trypsinogen, t-cell receptor excision circles)

2. Placental sample

DNA methylation profile

3. Newborn blood spot

DNA methylation

Metabolomic profile (Acyl-carnitines, amino acids, enzyme markers, haemoglobin profiles, hormones (thyroid stimulating hormone, 17-hydroxyprogesterone), immunoreactive trypsinogen, t-cell receptor excision circles)

DHEA-S, dehydroepiandrosterone-sulfate; DNA, Deoxyribonucleic acid; hCG, human chorionic gonadotrophin; hPL, human placental lactogen; LMP, PE(P-16:0e/0:0), 1-(1Z-hexadecenyl)-sn-glycero-3-phosphoethanolamine; PP, placental protein; SP1, pregnancy-specific beta-1-glycoprotein; THDOC, tetrahydrodeoxycorticosterone.

**Fig. 2:**
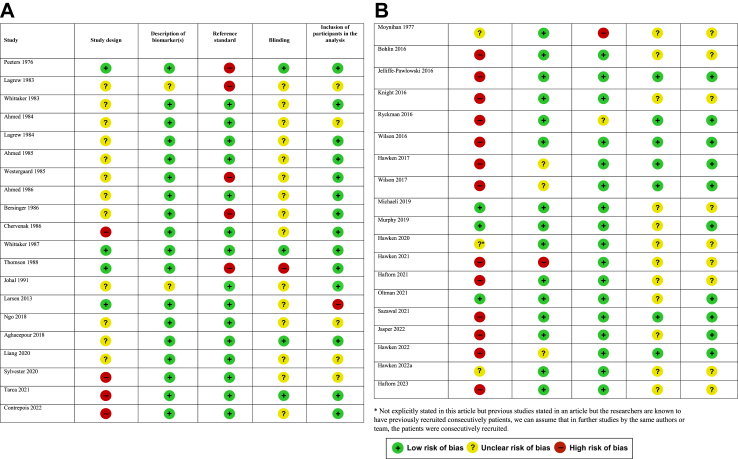
**A** Risk of bias assessment in studies assessing prenatal biomarkers. **B** Risk of bias assessment in studies assessing postnatal biomarkers.

The risk of bias in each included study is summarised in Fig. 2A (prenatal) and 2B (postnatal). Only one study^31^ was judged to be at low risk of bias for all five criteria (3%). Eight studies (21%) were deemed to be at low risk of bias for 4 domains and 14 were judged to be at low risk of bias for 3 domains (36%). The remaining 16 studies were judged to be at low risk of bias for ≤2 domains (41%). The most common shortcomings were related to the study design and blinding of researchers to the results of the “gold standard” GA. The majority of studies included in our review were prospective (56%), with 41% performed retrospectively and unclear in one study.^22^ Blinding of the gold standard was performed in 11 studies (28%), documented not to have occurred in one (3%) and unclear in the remaining 27 studies (69%). In 26 studies (67%) the reference GA was the gold standard (Ultrasound or LMP corroborated by ultrasound), 12 studies (33%) used LMP or ovulation date without ultrasound corroboration and one study (3%) used a combination of ovulation date with LMP and ultrasound.

### Accuracy of prenatal biomarkers to estimate gestational age

Overall, the correlation of the GA predicted by hCG in serum and the GA estimated by LMP was higher when testing was performed before 9 weeks than at or after 9 weeks (Table 1). Meta-analyses showed that the pooled coefficients of correlation were 0.88 (95% CI, 0.83–0.92, *I*^*2*^ = 56%; 6 studies) at 4–9 weeks and 0.43 (95% CI, 0.30–0.54, *I*^*2*^ = 0%; 3 studies) at 4–16 weeks (Table 3). For SP1, the pooled coefficients of correlation were 0.84 (95% CI, 0.80–0.88, I^2^ = 78%; 4 studies) at 5–10 weeks and 0.71 (95% CI, 0.66–0.75, I^2^ = 98%; 4 studies) at 5–16 weeks. For hPL, the pooled coefficient of correlation was 0.78 (95% CI, 0.69–0.84, *I*^*2*^ = 80%; 3 studies) at 6–16 weeks. The average difference between the GA predicted by hCG before 9 weeks and the GA estimated by LMP ranged between 0.4 and 2.2 weeks (median, 0.6 weeks; 5 studies).

**Table 3:** Meta-analyses of the predictive accuracy of prenatal and postnatal biomarkers for gestational age.

Biomarker	Biological sample	Population	Average deviation		Correlation coefficient			Predicted gestational age					
			Pooled RMSE (95% CI), weeks	No. of women or infants (No. of studies)	Pooled *r* (95% CI)	*I*^*2*^, %	No. of women or infants (No. of studies)	Within ±1 week of GA estimated by gold standard method			Within ±2 weeks of GA estimated by gold standard method		
								Pooled % (95% CI)	*I*^*2*^, %	No. of infants (No. of studies)	Pooled % (95% CI)	*I*^*2*^, %	No. of infants (No. of studies)
**Prenatal biomarkers**													
hCG between 4 and 9 weeks	Serum	Women	–	–	0.88 (0.83–0.92)	56	261 (6^22^, ^23^, ^24^, ^25^, ^26^, ^27^)	–	–	–	–	–	–
hCG between 4 and 16 weeks	Serum	Women	–	–	0.43 (0.30–0.54)	0	185 (3^22^^,^^25^^,^^27^)	–	–	–	–	–	–
SP1 between 5 and 10 weeks	Serum	Women	–	–	0.84 (0.80–0.88)	78	255 (4^24^^,^^26^^,^^27^^,^^29^)	–	–	–	–	–	–
SP1 between 5 and 16 weeks	Serum	Women	–	–	0.71 (0.66–0.75)	98	462 (4^24^^,^^27^^,^^29^^,^^32^)	–	–	–	–	–	–
hPL between 6 and 16 weeks	Serum	Women	–	–	0.78 (0.69–0.84)	80	303 (3^23^^,^^31^^,^^32^)	–	–	–	–	–	–
Metabolomic profile	Serum	Women	2.90 (2.61–3.21)	39 (2^37^^,^^38^)	0.90 (0.81–0.95)	0	39 (2^37^^,^^38^)	–	–	–	–	–	–
**Postnatal biomarkers**													
Metabolomic profile derived from newborn blood spot screening	Heel prick blood	All infants	1.03 (1.00–1.06)	536,612 (9^42^^,^^43^^,^^47^, ^48^, ^49^^,^^51^^,^^55^^,^^57^^,^^58^)	–	–	–	68.8 (65.8–72.0)	99.8	541,726 (11^42^^,^^43^^,^^47^, ^48^, ^49^^,^^51^, ^52^, ^53^^,^^55^^,^^57^^,^^58^^)^	94.4 (94.0–94.8)	91.6	488,028 (9^42^^,^^43^^,^^47^, ^48^, ^49^^,^^51^, ^52^, ^53^^,^^55^)
Metabolomic profile derived from newborn blood spot screening	Heel prick blood	Infants ≥37 weeks	1.00 (0.97–1.04)	102,244 (2^42^^,^^48^)	–	–	–	69.2 (69.0–69.3)	0	402,376 (3^42^^,^^47^^,^^48^)	96.1 (95.7–96.6)	96	402,376 (3^42^^,^^47^^,^^48^)
Metabolomic profile derived from newborn blood spot screening	Heel prick blood	Infants <37 weeks	1.56 (1.48–1.63)	107,627 (5^42^^,^^48^^,^^51^^,^^57^^,^^58^)	–	–	–	49.6 (39.0–63.1)	99.8	594,583 (10^42^^,^^44^^,^^47^^,^^48^^,^^51^^,^^52^^,^^57^^,^^58^)	80.1 (73.8–87.0)	98.9	590,687 (6^42^^,^^44^^,^^47^^,^^48^^,^^51^^,^^52^)
Metabolomic profile derived from newborn blood spot screening	Heel prick blood	Infants 32–36 weeks	–	–	–	–	–	50.1 (28.9–86.6)	100	544,942 (3^42^^,^^44^^,^^47^)	81.1 (69.7–94.4)	99.9	544,942 (3^42^^,^^44^^,^^47^)
Metabolomic profile derived from newborn blood spot screening	Heel prick blood	Infants <32 weeks	–	–	–	–	–	49.7 (46.4–53.2)	71.9	544,942 (3^42^^,^^44^^,^^48^)	76.6 (73.6–79.7)	75.4	544,942 (3^42^^,^^44^^,^^48^)
Metabolomic profile derived from newborn blood spot screening	Heel prick blood	SGA infants	1.43 (1.37–1.50)	185,018 (8^42^^,^^43^^,^^47^^,^^49^^,^^51^^,^^55^^,^^57^^,^^58^)	–	–	–	52.2 (45.8–59.4)	96.0	51,027 (7^48^^,^^49^^,^^51^, ^52^, ^53^^,^^55^^,^^57^^,^^58^)	86.9 (81.5–92.6)	94.8	48,791 (5^48^^,^^49^^,^^51^, ^52^, ^53^^,^^55^)
Metabolomic profile derived from cord blood samples	Cord blood	All infants	1.57 (1.03–2.39)	2198 (3^49^^,^^56^^,^^58^)	–	–	–	60.6 (59.2–62.1)	0	7345 (5^49^^,^^52^^,^^53^^,^^56^^,^^58^)	89.2 (87.0–91.6)	74.5	6333 (4^49^^,^^52^^,^^53^^,^^56^)
DNA methylation profile	Cord blood	All infants	1.60 (1.51–1.70)	183,042 (2^44^^,^^53^)	0.85 (0.78–0.89)	96.6	4501 (5^45^^,^^46^^,^^50^^,^^54^^,^^59^)	–	–		–	–	–

CI, confidence interval; GA, gestational age; hCG, human chorionic gonadotrophin; hPL, human placental lactogen; LMP, last menstrual period; *r*, correlation coefficient; RMSE, root mean square error; SP1, pregnancy-specific beta-1-glycoprotein.

The pooled coefficient of correlation for the GA predicted by metabolomic profiling in serum throughout the prenatal period and the GA estimated by ultrasound was 0.90 (95% CI, 0.81–0.95, *I*^*2*^ = 0%; 2 studies, 96 women). The pooled RMSE was 2.9 weeks (95% CI, 2.61–3.21). The AUC of models including several metabolites to discriminate between preterm birth and term birth was ∼ 0.90. One study^37^ reported that a model including 13 categories of metabolites predicted GA within 1 week of GA estimated by ultrasound for 67% of women and within 2 weeks for 78% of women. Two small studies reported that models of cell-free RNA transcripts^35^ and proteins^36^ in serum predicted GA with correlation coefficients of ∼0.90 and ∼0.95, respectively. Another study, involving 133 women, reported that whole-blood gene expression predicted GA with a correlation coefficient of 0.83 and a RMSE of 4.5 weeks.^39^

### Accuracy of postnatal biomarkers to estimate gestational age: metabolomic profile derived from newborn blood spot screening

Thirteen studies reported on the use of newborn blood spot to estimate GA. Overall, the average difference between GA predicted by the newborn metabolomic models and the GA estimated by LMP and/or ultrasound was slightly over 1 week (pooled RMSE of 1.03; 95% CI, 1.00–1.06; nine studies) (Table 3). This biomarker performed better among term infants (pooled RMSE of 1.00 week; 95% CI, 0.97–1.04; two studies) than among preterm infants (pooled RMSE of 1.56 weeks; 95% CI, 1.48–1.63; five studies) and SGA infants (pooled RMSE of 1.43 weeks; 95% CI, 1.37–1.50; eight studies).

Eleven studies provided data to calculate the ability of metabolomic profiling models to estimate GA within one and/or two week(s) of the gold standard using newborn blood spots. Overall, GA was correctly estimated by metabolomic profiling models to within one week of GA estimated by LMP and/or ultrasound for 68.8% of infants (95% CI, 65.8%–720%, *I*^*2*^ = 99.8%) and within 2 weeks for 94.4% of infants (95% CI, 94.0%–94.8%, *I*^*2*^ = 91.6%). Metabolomic profiling models performed best among term infants, correctly estimating GA to within one week in 69.2% of newborns (95% CI, 69.0%–69.3%, *I*^*2*^ = 0%; 3 studies) and within two weeks in 96.1% of newborns (95% CI, 95.7 to 96.6, *I*^*2*^ = 96%; 3 studies). The metabolomic profiling had a lower performance among infants born before 37 weeks (GA correctly estimated within one week in 49.6% and within two weeks in 80.1%), infants born between 32 and 36 weeks (GA correctly estimated within one week in 50.1% and within two weeks in 81.1%), infants born before 32 weeks (GA correctly estimated within one week in 49.7% and within 2 weeks in 76.6%), and SGA infants (GA correctly estimated within one week in 52.2% and within 2 weeks in 86.9%).

The pooled AUC of metabolomic profile derived from newborn blood spot screening to discriminate infants with a GA <37 weeks from those with a GA ≥37 weeks was 0.933 (95% CI, 0.905–0.962; *I*^*2*^ = 100%; 8 studies). The pooled AUC of metabolomic profile derived from newborn blood spot screening to discriminate infants with a GA <34 weeks from those with a GA ≥34 weeks was 0.986 (95% CI, 0.980–0.993; *I*^*2*^ = 91.2%; 3 studies).

### Accuracy of postnatal biomarkers to estimate gestational Age:Metabolomic profile derived from newborn cord blood

Overall, performance of metabolomic profiling models from cord blood was lower than that of metabolomic profiling models from samples of newborn heel pricks with a pooled RMSE of 1.57 weeks (95% CI, 1.03–2.39; three studies) and infants GAs correctly predicted within one week in 60.6% (95% CI, 59.2 to 62.1, *I*^*2*^ = 0%; 5 studies) and within two weeks in 89.2% (95% CI, 87.0 to 91.6, *I*^*2*^ = 74.5%; 4 studies). The pooled AUC of metabolomic profile models from cord blood in differentiating infants born before 37 weeks from those born at or after 37 weeks was 0.910 (95% CI, 0.873–0.946; *I*^*2*^ = 28%; 3 studies).^49^^,^^53^^,^^56^

### Accuracy of postnatal biomarkers to estimate gestational age: DNA methylation profile

The pooled average difference between GA predicted by cord blood DNA methylation profile and the GA estimated by LMP and ultrasound was 1.60 weeks (95% CI, 1.51–1.70; two studies) with a pooled coefficient of correlation of 0.85 (95% CI, 0.78 to 0.89, *I*^*2*^ = 96.6%; five studies).

### Exploration of heterogeneity

There was a significant level of heterogeneity among studies in the majority of meta-analyses performed. Most planned subgroup analyses could not be performed given the small number of studies included in the meta-analyses. However, subgroup analyses for maternal hCG between 4 and 9 weeks of gestation and the metabolomic profile derived from newborn blood spot screening in all infants, those born before <37 weeks of gestation, and those born SGA showed that the study setting, sample size, and the study quality did not provide an explanation for heterogeneity.

## Discussion

Metabolomic profiling from newborn heel prick blood spots during the immediate postnatal period provided the most accurate estimate of GA, with a pooled RMSE of 1.03 weeks across all GAs. It performed best for term infants, showing slightly reduced accuracy for preterm or SGA infants. In addition, the metabolomic profile derived from newborn blood spot screening appeared to differentiate between preterm (<37 weeks) and term (≥37 weeks) infants, as well as between infants born before 34 weeks and those at or after 34 weeks with a pooled AUC of 0.93 and 0.98, respectively.

Metabolomic profiling and DNA methylation profile from cord blood samples provided less accurate estimates of GA compared to metabolomic profiling from heel prick blood samples, with pooled RMSEs of 1.57 and 1.60 weeks, respectively.

Among the placental hormones assessed, hCG measured between 4 and 9 weeks of gestation showed the highest correlation with the reference standard GA, usually estimated by LMP, with a pooled coefficient of correlation of 0.88. However, the pooled RMSE could not be estimated due to lack of data.

Evidence from two small studies indicated that metabolomic profiling from maternal blood samples collected throughout pregnancy to estimate GA had low accuracy, with a pooled RMSE of 2.90 weeks.

Insufficient evidence was available to evaluate other prenatal biomarkers, such as cell-free RNA transcripts, proteomic profile, and exon-level gene expression.

Our systematic review has identified promising methods for postnatal GA estimation using algorithms that combine metabolomic profile derived from newborn heel-prick blood spots with clinical and demographic variables (mainly birthweight, sex, and multiple birth status). These algorithms estimated GA postnatally to within approximately 1 week of a reference standard. This approach outperformed neonatal assessments like the Dubowitz and Ballard scores, which deviate by ±2.6 to 3.8 weeks from the gold standard GA.^12^ Therefore, metabolomics modelling based on heel-prick blood spot samples are highly likely to be a more accurate way to estimate GA when early pregnancy ultrasound is not available. Importantly, the metabolomic profile derived from newborn blood spot showed consistent performance across a variety of settings and ethnicities, including in high-income countries and LMICs. However, the requirement of tandem mass spectrometers or other necessary devices required for metabolomic profiling from heel prick blood spots poses a major limitation for implementation, particularly in resource-poor settings, and the cost of testing, estimated at approximately USD $50 per child, is also a significant obstacle.^60^

The second major limitation is that postnatal GA estimation using metabolomic profiling from cord blood samples or heel prick blood spots is not available to guide clinical prenatal care at the level of the individual woman. Therefore postnatal markers are not useful for prenatal care. Nevertheless, postnatal markers could be a fruitful avenue to guide research if they can be assessed in maternal blood or urine prenatally.

The challenges and importance of improving pathology and laboratory provision in LMICs was discussed in the Lancet series.^61^, ^62^, ^63^ The authors identified four key barriers to achieving optimal laboratory services in LMICs including lack of: trained personnel, education and training, infrastructure and agreed quality standards and accreditation.^63^ Costs are lower if the turn-around time is longer and if the laboratory is performing higher numbers of tests^61^ which could be overcome by centralising resources, however, this would prevent the results being available for individualised care of the pregnant woman or neonate.

There were differences in accuracy of postnatal GA estimation between metabolomic profiles derived from cord blood samples and heel-prick blood spots. In theory, most proteins or transcripts can be assessed in numerous different samples, and such differences may simply be due to variations in techniques rather than whether they are coming from cord or heel-prick. However, differences could also be attributable to various other factors, including timing of collection, fluctuations in neonatal analyte levels during the early postpartum period, and infant feeding status prior to collection.^49^ Samples taken directly from the newborn may better reflect their physiology compared to cord specimens. Although metabolomic profiling derived from cord blood samples for estimating GA was less accurate than from heel prick blood samples, it still outperformed Dubowitz and Ballard scores. Collecting cord blood samples does not cause discomfort to the newborn, may be more acceptable to parents and avoids extensive training required for heel prick sample collection techniques.

The main strengths of our study include the following: (1) the rigorous methodology used for performing the systematic review; (2) the use of a prospective protocol designed to answer a specific research question; (3) the extensive and continually updated literature searches without language restrictions; (4) the strict assessment of the risk of bias of the included studies; (5) the quantitative way of summarizing the evidence; and (6) the inclusion of >1.7 million newborns in the studies that examined the accuracy of postnatal biomarkers. Some potential limitations must also be considered. First, there was an important degree of heterogeneity in most of the meta-analyses performed. We explored the sources of heterogeneity and were unable to identify plausible explanations; therefore, pooled estimates should be interpreted cautiously. We used a random-effects model to pool results from individual studies, which provides the most useful and conservative estimate for informing practice in the presence of unexplained heterogeneity. Nevertheless, the between-study heterogeneity, inability to assess publication bias and small number of studies remain an important limitation of the study. Second, study quality was a limitation of the studies included in the review with only one-fourth of included studies being judged to be at low risk of bias for at least four domains. Third, most studies that assessed placental hormones did not use an appropriate reference standard for pregnancy dating because ultrasound was not widely available when such studies were conducted. Moreover, there was heterogeneity of reference ultrasound timing in some studies assessing postnatal biomarkers, mainly those conducted in LMICs, in which only a small proportion of women had reference ultrasound completed between 9 and 13 weeks of gestation. Fourth, authors of included studies chose a wide variety of statistics to report accuracy of biomarkers to estimate GA, which made combining results difficult. Fifth, the use of the logit transformation approach and ignorance of population weights in the calculation of pooled proportions has the potential to produce misleading pooled estimates. Sixth, we used the DerSimonian and Laird approach for random-effects meta-analyses, and it is possible that the 95% CIs of our meta-analyses could be slightly different if other statistical methods proposed for adjusting them are used (such as the Hartung, Knapp, Sidik and Jonkman or modified Knapp−Hartung methods for random effects meta-analysis). However, the approach we use is recommended in Cochrane reviews and these methods differ only in respect to the calculation of the confidence intervals, not pooled estimates. Finally, the number of studies that assessed several biomarkers, mainly prenatal ones, is still too small for us to draw firm conclusions.

In conclusion, our study has identified several candidate biomarkers that could estimate GA in settings where early ultrasound is unavailable. Further studies are required to compare the most promising of these biomarkers to each other, as well as to other modalities such as ultrasound, fundal height or other clinical markers of GA assessment, in order to identify which will prove most useful. Several factors would need to be considered including accuracy, cost, equipment and infrastructure required. This is particularly important in LMIC settings in which pregnancy dating is especially challenging. Cultural acceptability of such a test would also be an important consideration, as we know parents may prefer a cord blood sample to be obtained over a heel prick blood sample.^51^ Therefore, cord blood tests may have a place despite their lower accuracy in estimating GA. Finally, simplification of metabolomic profiling models to reduce the number of analytes while maintaining a good accuracy to estimate GA will be required to streamline the approach for scalable, cost-effective applications. Thus, although -omics technology is too expensive and impractical for widespread use, the techniques can be used to identify proteins of interest. In turn, inexpensive, point of care assays could then be developed for these proteins. Future studies should report on cost, as these methods are likely to have most benefit in LMIC settings where cost is an important barrier to implementation.

## Contributors

ATP: Funding acquisition, project administration; EB, ATP: Conceptualisation; EB, ACA, NR, JV, ATP: Design of methodology; NR, EB, ACA: Literature search; ACA, EB: Data analysis; ACA, EB, JV, ATP: Data interpretation; EB, ACA, ATP: Writing–original draft; All authors: Writing—review & editing,: all authors read and approved the final version of the manuscript. ATP, EB and ACA have directly accessed and verified the underlying data reported in the manuscript.

## Data sharing statement

The data in this publication are drawn exclusively from publicly available sources and datasets. All relevant information, including aggregated data and key study characteristics, can be accessed in the original publications cited in this review.

## Declaration of interests

A.T.P. is a Senior Advisor of Intelligent Ultrasound. ATP is supported by the Oxford Partnership Comprehensive Biomedical Research Centre with funding from the NIHR Biomedical Research Centre (BRC) funding scheme. The views expressed herein are those of the authors and not necessarily those of the NHS, the NIHR the Department of Health or any of the other funders. All other authors declare no competing interests.

## Appendix A Supplementary data

Search strategies
