# Supplementary material for: Accuracy of prenatal and postnatal biomarkers for estimating gestational age: a systematic review and meta-analysis
Source: eClinicalMedicine. 2024 Mar 8;70:102498. doi: 10.1016/j.eclinm.2024.102498 (PMC10940947; doi:10.1016/j.eclinm.2024.102498)
Supplement: Search strategies [file mmc1.docx]

Appendix: Searches

|  | [Medline (Ovid MEDLINE® Epub Ahead of Print, In-Process & Other Non-Indexed Citations, Ovid MEDLINE® Daily and Ovid MEDLINE®) 1946 to present](https://ovidsp.ovid.com/ovidweb.cgi?T=JS&NEWS=N&PAGE=main&SHAREDSEARCHID=3iXtn5TAjGPmKThCWyQPeWTbWNKLajR0g3VmMIZDjfh6BprwHqVUuuhRQjocAw1RT) |
| --- | --- |
| 1 | Gestational Age/ |
| 2 | (gestation* adj age).mp. |
| 3 | 1 or 2 |
| 4 | ((marker? or biomarker?) adj10 (predict* or correlat* or determin* or assess* or date or dating)).mp. |
| 5 | ((predict* or correlat* or determin* or assess* or date or dating) adj2 (tool? or model* or algorithm*)).mp. |
| 6 | 4 or 5 |
| 7 | 3 and 6 |
| 8 | ((predict* or correlat* or determin* or assess* or date or dating) adj5 (gestation* adj age)).mp. |
| 9 | ((predict* or correlat* or determin* or assess* or date or dating) and gestation* and age).ti. |
| 10 | 7 or 8 or 9 |
| 11 | exp "reproducibility of results"/ or exp "sensitivity and specificity"/ |
| 12 | (sensitiv* or specific* or accura* or reproduc* or validat* or predictive value or ppv or npv).mp. |
| 13 | 11 or 12 |
| 14 | 10 and 13 |
| 15 | exp animals/ not humans/ |
| 16 | 14 not 15 |
| 17 | (comment or editorial or letter or news or "review" or case report).pt. or case report.tw. |
| 18 | 16 not 17 |
| 19 | limit 16 to ("systematic review" and "reviews (maximizes specificity)") |
| 20 | 18 or 19 |

|  | [Embase (OvidSP) 1974 to present](https://ovidsp.ovid.com/ovidweb.cgi?T=JS&NEWS=N&PAGE=main&SHAREDSEARCHID=1MuJTRyM9KGAIsPxJZzTwiEOwBfoDEyMjhZQQJjRQHI7pTsihDiSeoHOsucGARCuR) |
| --- | --- |
| 1 | Gestational Age/ |
| 2 | (gestation* adj age).mp. |
| 3 | 1 or 2 |
| 4 | ((marker? or biomarker?) adj10 (predict* or correlat* or determin* or assess* or date or dating)).mp. |
| 5 | ((predict* or correlat* or determin* or assess* or date or dating) adj2 (tool? or model* or algorithm*)).mp. |
| 6 | 4 or 5 |
| 7 | 3 and 6 |
| 8 | ((predict* or correlat* or determin* or assess* or date or dating) adj5 (gestation* adj age)).mp. |
| 9 | ((predict* or correlat* or determin* or assess* or date or dating) and gestation* and age).ti. |
| 10 | 7 or 8 or 9 |
| 11 | exp "reproducibility of results"/ or exp "sensitivity and specificity"/ |
| 12 | (sensitiv* or specific* or accura* or reproduc* or validat* or predictive value or ppv or npv).mp. |
| 13 | 11 or 12 |
| 14 | 10 and 13 |
| 15 | (exp animals/ or nonhuman/) not human/ |
| 16 | 14 not 15 |
| 17 | (editorial or letter or note or press or "review").pt. or case report/ or case report.tw. |
| 18 | 16 not 17 |
| 19 | limit 16 to ("systematic review" and "reviews (maximizes specificity)") |
| 20 | 18 or 19 |

Science Citation Index & Conference Proceedings Citation Index – Science (Web of Science Core Collection)[1900-present]

| # 13 | #12 OR #10 |
| --- | --- |
| # 12 | #11 AND #9 |
| # 11 | TS=("systematic review" OR "systematic literature review" OR meta-analys* OR "meta analys*" OR metaanalys*) |
| # 10 | #7 NOT #8 Refined by: [excluding] DOCUMENT TYPES: ( EDITORIAL MATERIAL OR LETTER OR NOTE OR REVIEW ) |
| # 9 | #7 NOT #8 |
| # 8 | TS=(animals or animal or mice or mus or mouse or murine or woodmouse or rats or rat or murinae or muridae or cottonrat or cottonrats or hamster or hamsters or cricetinae or rodentia or rodent or rodents or pigs or pig or swine or swines or piglets or piglet or boar or boars or sus scrofa or ferrets or ferret or polecat or polecats or mustela putorius or guinea pigs or guinea pig or cavia or callithrix or marmoset or marmosets or cebuella or hapale or octodon or chinchilla or chinchillas or gerbillinae or gerbil or gerbils or jird or jirds or merione or meriones or rabbits or rabbit or hares or hare or diptera or flies or fly or dipteral or drosphila or drosophilidae or cats or cat or carus or felis or nematoda or nematode or nematoda or nematode or nematodes or sipunculida or dogs or dog or canine or canines or canis or sheep or sheeps or mouflon or mouflons or ovis or goats or goat or capra or capras or rupicapra or chamois or haplorhini or monkey or monkeys or anthropoidea or anthropoids or saguinus or tamarin or tamarins or leontopithecus or hominidae or ape or apes or pan or paniscus or pan paniscus or bonobo or bonobos or troglodytes or pan troglodytes or gibbon or gibbons or siamang or siamangs or nomascus or symphalangus or chimpanzee or chimpanzees or prosimians or bush baby or prosimian or bush babies or galagos or galago or pongidae or gorilla or gorillas or pongo or pygmaeus or pongo pygmaeus or orangutans or pygmaeus or lemur or lemurs or lemuridae or horse or horses or pongo or equus or cow or calf or bull or chicken or chickens or gallus or quail or bird or birds or quails or poultry or poultries or fowl or fowls or reptile or reptilia or reptiles or snakes or snake or lizard or lizards or alligator or alligators or crocodile or crocodiles or turtle or turtles or amphibian or amphibians or amphibia or frog or frogs or bombina or salientia or toad or toads or epidalea calamita or salamander or salamanders or eel or eels or fish or fishes or pisces or catfish or catfishes or siluriformes or arius or heteropneustes or sheatfish or perch or perches or percidae or perca or trout or trouts or char or chars or salvelinus or fathead minnow or minnow or cyprinidae or carps or carp or zebrafish or zebrafishes or goldfish or goldfishes or guppy or guppies or chub or chubs or tinca or barbels or barbus or pimephales or promelas or poecilia reticulata or mullet or mullets or seahorse or seahorses or mugil curema or atlantic cod or shark or sharks or catshark or anguilla or salmonid or salmonids or whitefish or whitefishes or salmon or salmons or sole or solea or sea lamprey or lamprey or lampreys or pumpkinseed or sunfish or sunfishes or tilapia or tilapias or turbot or turbots or flatfish or flatfishes or sciuridae or squirrel or squirrels or chipmunk or chipmunks or suslik or susliks or vole or voles or lemming or lemmings or muskrat or muskrats or lemmus or otter or otters or marten or martens or martes or weasel or badger or badgers or ermine or mink or minks or sable or sables or gulo or gulos or wolverine or wolverines or minks or mustela or llama or llamas or alpaca or alpacas or camelid or camelids or guanaco or guanacos or chiroptera or chiropteras or bat or bats or fox or foxes or iguana or iguanas or xenopus laevis or parakeet or parakeets or parrot or parrots or donkey or donkeys or mule or mules or zebra or zebras or shrew or shrews or bison or bisons or buffalo or buffaloes or deer or deers or bear or bears or panda or pandas or wild hog or wild boar or fitchew or fitch or beaver or beavers or jerboa or jerboas or capybara or capybaras) |
| # 7 | #6 AND #5 |
| # 6 | TS=(sensitiv* or specific* or accura* or reproduc* or validat* or predictive value or ppv or npv) |
| # 5 | #4 OR #3 |
| # 4 | TS=((predict* or correlat* or determin* or assess* or date or dating) NEAR/5 ("gestational age" OR "gestation age")) OR TI=((predict* or correlat* or determin* or assess* or date or dating) and gestation* and age) |
| # 3 | #2 AND #1 |
| # 2 | TS=((marker* or biomarker*) NEAR/10 (predict* or correlat* or determin* or assess* or date or dating)) OR TS=((predict* or correlat* or determin* or assess* or date or dating) NEAR/2 (tool* or model* or algorithm*)) |
| # 1 | TS=(gestation* NEAR/2 age) |

CINAHL (EBSCOHost)[1982-present]

| Search ID# | Search Terms |
| --- | --- |
| S17 | S14 OR S16 |
| S16 | S13 NOT S15 |
| S15 | S9 AND S12 Limiters - Publication Type: Editorial, Letter, Review |
| S14 | S9 AND S12 Limiters - Clinical Queries: Review - High Specificity |
| S13 | S9 AND S12 |
| S12 | S10 OR S11 |
| S11 | TI ( (sensitiv* or specific* or accura* or reproduc* or validat* or predictive value or ppv or npv) ) OR AB ( (sensitiv* or specific* or accura* or reproduc* or validat* or predictive value or ppv or npv) ) |
| S10 | (MH "Sensitivity and Specificity") OR (MH "Predictive Value of Tests") |
| S9 | S7 OR S8 |
| S8 | AB ( ((predict* or correlat* or determin* or assess* or date or dating) N5 (gestation* N1 age)) ) OR TI ( (predict* or correlat* or determin* or assess* or date or dating) AND gestation* AND age ) |
| S7 | S3 AND S6 |
| S6 | S4 OR S5 |
| S5 | TI ( ((predict* or correlat* or determin* or assess* or date or dating) N2 (tool* or model* or algorithm*)) ) OR AB ( ((predict* or correlat* or determin* or assess* or date or dating) N2 (tool* or model* or algorithm*)) ) |
| S4 | TI ( ((marker* or biomarker*) N10 (predict* or correlat* or determin* or assess* or date or dating)) ) OR AB ( ((marker* or biomarker*) N10 (predict* or correlat* or determin* or assess* or date or dating)) ) |
| S3 | S1 OR S2 |
| S2 | TI (gestation* N1 age) OR AB (gestation* N1 age) |
| S1 | (MH "Gestational Age") |
